# Inequalities in Care-seeking for Febrile Illness of Under-five Children in Urban Dhaka, Bangladesh

**DOI:** 10.3329/jhpn.v29i5.8907

**Published:** 2011-10

**Authors:** Nusrat Najnin, Catherine M. Bennett, Stephen P. Luby

**Affiliations:** ^1^icddr,b, GPO Box 128, Dhaka 1000, Bangladesh; ^2^Melbourne School of Population Health, University of Melbourne, Melbourne, Australia; ^3^School of Health and Social Development, Deakin University, Melbourne, Australia

**Keywords:** Cross-sectional studies, Fever, Gender identity, Health personnel, Patient acceptance of healthcare, Socioeconomic factors, Urban population, Bangladesh

## Abstract

Fever is an easily-recognizable primary sign for many serious childhood infections. In Bangladesh, 31% of children aged less than five years (under-five children) die from serious infections, excluding confirmed acute respiratory infections or diarrhoea. Understanding healthcare-seeking behaviour for children with fever could provide insights on how to reduce this high rate of mortality. Data from a cross-sectional survey in the catchment areas of two tertiary-level paediatric hospitals in Dhaka, Bangladesh, were analyzed to identify the factors associated with the uptake of services from trained healthcare providers for under-five children with reported febrile illness. Health and demographic data were collected in a larger study of 7,865 children using structured questionnaires. Data were selected from 1,290 of these under-five children who were taken to any healthcare provider for febrile illness within two months preceding the date of visit by the study team. Certified doctors were categorized as ‘trained’, and other healthcare providers were categorized as ‘untrained’. Healthcare-seeking behaviours were analyzed in relation to these groups. A wealth index was constructed using principal component analysis to classify the households into socioeconomic groups. The odds ratios for factors associated with healthcare-seeking behaviours were estimated using logistic regression with adjustment for clustering. Forty-one percent of caregivers (n=529) did not seek healthcare from trained healthcare providers. Children from the highest wealth quintile were significantly more likely [odds ratio (OR)=5.6, 95% confidence interval (CI) 3.4-9.2] to be taken to trained healthcare providers compared to the poorest group. Young infants were more likely to be taken to trained healthcare providers compared to the age-group of 4-<5 years (OR=1.6, 95% CI 1.1-2.4). Male children were also more likely to be taken to trained healthcare providers (OR=1.5, 95% CI 1.2-1.9) as were children with decreased level of consciousness (OR=5.3, 95% CI 2.0-14.2). Disparities across socioeconomic groups and gender persisted in seeking quality healthcare for under-five children with febrile illness in urban Dhaka. Girls from poor families were less likely to access qualified medical care. To reduce child mortality in the short term, health education and behaviour-change communication interventions should target low-income caregivers to improve their recognition of danger-signs; reducing societal inequalities remains an important long-term goal.

## INTRODUCTION

Bangladesh has achieved significant success in reducing mortality of children aged less than five years (under-five children) over the past few decades but this rate remains high at 65 per 1,000 livebirths (1). Data of the 2004 Bangladesh Demographic and Health Survey (BDHS) identified the cause of death of 31% of urban under-five children as serious infections other than confirmed acute respiratory infections (ARIs) or diarrhoea (2). For many serious infections, including typhoid fever, bactaerimia, septicaemia, and meningo-encephalitis, fever is an easily-recognizable primary sign.

Two important factors that contribute to the high rate of child mortality in low-income countries are a low rate of healthcare-seeking from trained healthcare providers and a delay in seeking this care (3-6). Arifeen and colleagues have suggested that one way to reduce child mortality in urban Bangladesh would be by improving healthcare-seeking patterns of caregivers of under-five children (7).

Several other studies have been conducted in Bangladesh to understand care-seeking behaviour for child health but most of these were related to ARIs and were undertaken in rural areas (7-10). Limited information is available on the factors that determine healthcare-seeking behaviour for children from trained providers with febrile illness in urban settings. This is especially important as metropolitan Dhaka, with a population of about 10 million, continues to grow at a rate of 320,000 people per year (11-12).

This paper presents an analysis of data collected within a larger study that had a broader aim of evaluating the burden of vaccine-preventable diseases in the catchment areas of two hospitals in urban Dhaka (13). This analysis of data intended to identify the specific factors that could affect seeking healthcare for under-five children with reported febrile illness from trained healthcare providers in an urban setting where qualified healthcare provision is not a key limiting factor. The study aims to recommend steps that could help prevent health consequences from serious infections due to lack of appropriate healthcare.

## MATERIALS AND METHODS

### Study population and data-collection

The Dhaka Shishu Hospital and the Shishu Sasthya Foundation Hospital are two tertiary-level paediatric hospitals in metropolitan Dhaka having both inpatient and outpatient services provided by qualified medical doctors (13). Both the hospitals provide free inpatient services to 20-48% of their patients. But the cost for an outpatient consultation ranges from US$ 0.30 to US$ 7.00.

As part of community assessment of the larger study, icddr,b field researchers at the Dhaka Shishu Hospital and Shishu Sasthya Foundation Hospital prepared daily lists of the 20 most recent admissions of under-five children who had been diagnosed with pneumonia, meningitis, sepsis, or presumed enteric fever (13). The patient lists were prepared during August-October 2007, and the project kept a record of the addresses of the enlisted patients. The study team used a random number table to select patients from the lists of both the hospitals in numbers proportional to the number of children admitted daily to these hospitals from a distance of within 60-minute travel time. The travel time cut-off was specified to define the hospital catchment areas so that the larger study could focus on those people who used the Dhaka Shishu Hospital and the Shishu Sasthya Foundation Hospital as their regular source of care. This precise 60-minute travel time cut-off was chosen after discussion with collaborators at the Dhaka Shishu Hospital whose impression was that patients who use the hospital as their primary source of care generally lived within 60-minute travel time.

A researcher visited the household of each patient to confirm that it was within the travel time cut-off and identified the geographically-closest sixth household to enroll any under-five child or any under-five child who had died within the last one year. All the under-five children within a household were eligible to be enrolled in the larger study. The study team continued to move forward to the next closest households in each of the two hospital catchment areas and enrolled 7,865 children from 70 clusters, each cluster consisting of 100 households.

Two structured questionnaires—household questionnaire and child questionnaire—were used in face-to-face interviews with the caregivers. The household questionnaire collected basic demographic and socioeconomic information. The study team also collected information on type of household construction, household assets, and possessions and monthly income of households in Bangladeshi taka, along with the educational and employment status of parents. Then a child questionnaire was administered for each under-five child to collect information on healthcare-seeking from the provider for any febrile and respiratory illness within the last two months. The interviewers specifically asked the caregivers if the child had fever, cough, difficulty in breathing, or in staying awake during the illness episode for which the child was taken to a healthcare provider.

### Children with febrile illness

For this paper, we analyzed the data of those under-five children whose caregivers reported that they had been taken to a healthcare provider with symptoms of febrile illness but without symptoms of respiratory illness, such as cough or difficulty in breathing, within two months of the date of interview.

### Healthcare providers

We categorized the healthcare providers who gave services in our two hospital catchment areas into three main groups: trained, traditional, and untrained. Doctors with MBBS degree were categorized as trained; herbalists and homeopaths were considered traditional; and providers who did not have any such training or experience were considered untrained, e.g. paramedics and drug-sellers. For multiple regression analyses, traditional and untrained care providers were grouped as untrained healthcare providers.

### Analysis of data

We constructed a wealth index using principal component analysis based on the presence or absence of items from a list of household assets or possessions (14). The wealth index included type of household construction and 14 household assets or possessions, including commodities that are commonly used in Bangladesh and are considered to be discriminatory (15). To construct the wealth index, we followed a methodology similar to that used for creating the wealth index for urban Dhaka previously in a same study population (16). The wealth index allocated the highest score to the more-affluent households that had a refrigerator and the lowest to households that did not own a sewing machine. The variance of the first principal component was 8.5 which explained 60% of the total variance. The households were divided into wealth quintiles based on their scores.

We used the Stata statistical software (version 10) to analyze data. We estimated the odds ratios and 95% confidence limits for factors associated with healthcare-seeking behaviours using logistic regression. We had 70 clusters from the hospital catchment areas (catchment areas were defined based on the 60-minute travel time cut-off) in our study. It is possible that healthcare-seeking behaviour within one cluster was similar but that between clusters it could vary. We considered this in our analysis by using sandwich style estimators for deriving the cluster-adjusted standard errors to account for the residual correlation due to repeated measures (17).

### Model and variable specification

The primary outcome variable was seeking healthcare from a trained healthcare provider. Whenever a caregiver consulted a trained healthcare provider, irrespective of his/her consultation with an untrained provider, we considered it as seeking care from a trained provider. The independent variables were household wealth index, age and gender of the child, educational status of the father and the mother, and presence of decreased level of consciousness along with fever. The educational status of both father and mother was associated with seeking care from a trained provider in the univariate logistic regression analysis. However, we excluded education of mothers from the multiple regression model as it had a high correlation with education of fathers (correlation coefficient 0.8), and education of fathers was a better of the two as predictor of care-seeking behaviour. Each independent variable was evaluated for confounding and effect modification.

### Ethical considerations

Informed written consent was obtained from the adult study participant from each household. Confidentiality of data was maintained throughout the study period and during analysis. The Ethical Review Committee of icddr,b reviewed and approved the study protocol.

## RESULTS

In total, 7,865 children were enrolled in the larger study. From these, we first selected 1,471 children who had been seen by a healthcare provider for a febrile illness within the past two months preceding the date of visit by the study team. One hundred sixty-six households had more than one under-five child. The intraclass correlation coefficient of children within these households was 0.5, indicating that the children from the same household had comparable characteristics. We identified these households and selected a single child randomly from each household until we could select 1,290 children as study participants. The proportion of the hospital catchment areas included in the main study was also applied to this sub-sample of 1,290 children extracted for febrile illness. Fifty-seven percent (n=730) of these participants were from the Shishu Sasthya Foundation Hospital catchment area, and 43% (n=560) were from the Dhaka Shishu Hospital catchment area.

Of the study participants, 54% were males and 41% were aged less than two years. Twenty-six percent of mothers (n=334) and 19% of fathers (n=249) did not finish primary school. Twenty-three percent of parents earned approximately US$ 73 (Tk 5,000) per month (Table 1).

While 59% (n=761) of the study population sought care from the trained healthcare providers for febrile illness, 35% (n=452) sought it from the untrained providers, and 6% (n=77) from the traditional care providers. Only 13% (n=163) sought care from the outpatient department of the study hospitals. A child was significantly more likely to be seen by a trained healthcare provider if the parents were wealthier, if the father had finished secondary school, if the child was a male, if the child had decreased level of consciousness, and if the child aged less than two years (Table 2).

Table 3 compares the findings of univariate and multiple logistic regression analyses, examining the factors associated with taking a child to see a trained healthcare provider. The importance of education of father diminished when adjusted for wealth and age and illness characteristics of the child. We tested for interaction for each of the independent variables and found none.

**Table 1.** Sociodemographic characteristics of study participants

Characteristics	No.	% *
No. of children	1,290	
Age (years) of children		
<1	224	17
1-<2	303	24
2-<3	239	19
3-<4	251	19
4-<5	273	21
Gender		
Male	695	54
Education of mothers		
No schooling and some primary	334	26
Finished primary and some secondary	493	38
Finished secondary	459	36
Missing	4	0.3
Education of fathers		
No schooling and some primary	249	19
Finished primary and some secondary	406	31
Finished secondary	622	48
Missing	13	1
Occupation of fathers		
Salaried employee	652	50
Shopkeeper/merchant	331	26
Employed on daily wages	220	17
Other	70	6
Unemployed	15	1
Unknown	2	0.2
Monthly household income (US$) †	
<29-73	302	23
>73-145	482	38
>145 and above	503	39
Unknown	3	0.2
Household assets		
Computer	130	10
Television (colour)	694	54
Refrigerator	513	40
Sewing machine	228	18
Motor cycle	49	4
Mobile phone	943	73
Land phone	121	9
Blanket	616	48
Bed (*khat*)	1,065	83
Car/truck	37	3
Construction of house and available facility		
Tin roof	679	53
Brick wall	1,148	89
Cement floor	1,229	95
Natural gas connection for cooking	1,192	93

* Some categories do not sum to 100% because of rounding;

† Based on the 2007 exchange rate (US$ 1=Tk 69)

Children living in households in the highest wealth quintile (fifth quintile) were significantly more likely to be taken to trained healthcare providers compared to the poorest group—quintile 1 [odds ratio (OR)=5.6, 95% confidence interval (CI) 3.4-9.2**]**. On exploratory analysis of the wealth index, we found that the people from the poorest quintile had incomes of less than US$ 1 per person per day.

The higher the educational status of the father, the more likely that the child was taken to a trained healthcare provider (OR=1.8, 95% CI 1.2-2.5). The male children were 50% more likely to be taken to trained healthcare providers (OR=1.5, 95% CI 1.2-1.9), and children with decreased level of consciousness were also more likely (OR=5.3, 95% CI 2.0-14.2) to be seen by a trained healthcare provider (Table 3). The younger the children, the more likely that they were taken to a trained provider (OR=1.6, 95% CI 1.1-2.4).

Of all the caregivers who sought care from any healthcare providers, 80% (n=1,031) did so within 48 hours of onset of febrile illness. However, 42% (n=437) of these caregivers who sought help within 48 hours did so from either unqualified or traditional providers (Fig.). We chose 48 hours as the cut-off time for seeking care as any longer was well above the median duration of the illness before healthcare was sought outside the home. Only 58% of children with a febrile illness were brought to a trained healthcare provider within 48 hours. The proportion of seeking care from a trained healthcare provider within 48 hours of onset of fever was higher (72%, n=21) if a child had a decreased level of consciousness.

## DISCUSSION

We identified specific factors linked to socioeconomic status, gender, and perceived vulnerability associated with the uptake of services from trained healthcare providers for under-five children with reported febrile illness. Of the study participants, children from the poorest families were least likely to seek care from trained healthcare providers; boys were more likely to be taken to trained healthcare providers; and a decreased level of consciousness acted as trigger for carers to seek healthcare from trained healthcare providers.

**Table 2.** Proportion of children with febrile illness seeking care from different healthcare providers*

Sociodemographic and illness characteristics	Children with fever who sought any care (No.)	Types of healthcare providers			Sought care from >1 provider for febrile illness %(95% CI)
	Trained % (95% CI)	Untrained % (95% CI)	Traditional % (95% CI)		
All children	1,290	59 (55-63)	35 (32-39)	6 (4-7)	2 (2-3)
Age (years) of children					
<1	224	64 (57-71)	25 (19-31)	11 (7-15)	3 (1-5)
1-<2	303	66 (60-72)	30 (24-36)	4 (1-6)	3 (1-5)
2-<3	239	59 (51-66)	36 (29-44)	5 (2-8)	1 (0.1-3)
3-<4	251	52 (45-58)	44 (38-51)	4 (1-7)	4 (1-6)
4-<5	273	54 (46-61)	41 (34-48)	6 (3-8)	2 (0.2-3)
Presence of decreased level of consciousness					
Yes	34	85 (73-98)	9 (-0.01-18)	6 (2-13)	12 (0.01-24)
Sex					
Male	695	62 (58-66)	33 (29-37)	5 (3-7)	3 (2-4)
Female	595	55 (50-60)	39 (34-43)	6 (4-8)	2 (1-3)
Education of mothers					
No schooling and some primary	334	44 (37-51)	51 (45-57)	5 (3-8)	3 (1-5)
Finished primary and some secondary	493	56 (51-61)	39 (34-43)	6 (3-8)	3 (2-5)
Finished secondary	459	73 (68-77)	21 (17-25)	6 (4-9)	2 (0.3-3)
Education of fathers					
No schooling and some primary	249	40 (33-48)	55 (48-63)	4 (2-7)	4 (2-7)
Finished primary and some secondary	406	52 (47-57)	41 (36-47)	6 (4-9)	3 (1-5)
Finished secondary	622	71 (67-75)	23 (19-27)	6 (4-8)	1 (1-2)
Socioeconomic status (by quintile)					
First (poorest)	219	35 (26-43)	59 (51-68)	6 (2-10)	4 (1-7)
Second	266	48 (42-55)	45 (39-51)	6 (3-10)	2 (1-4)
Third	247	60 (53-66)	35 (29-41)	6 (3-9)	2 (1-4)
Fourth	267	65 (60-71)	30 (25-35)	5 (2-8)	3 (1-5)
Fifth (richest)	223	81 (75-86)	14 (10-19)	5 (2-8)	2 (0.1-3)

* The proportions and 95% confidence intervals have been corrected for the cluster design of the study

Socioeconomic status has previously been identified as a strong predictor of healthcare-seeking behaviour for under-five children, not only in Bangladesh but also in many other low-income countries (18-22). According to the wealth index of our study, the poorest group was five times less likely to seek quality care compared to the richest group. These findings are consistent with findings of studies focusing on diarrhoea and ARI in rural Bangladeshi communities (7,15,23). Although urban Dhaka has a plethora of private health physicians and facilities, the lack of access to a trained healthcare provider due to inequity in healthcare-seeking by socioeconomic status remains a barrier to the pathway of achieving a better health outcome for the poor and reducing child mortality (1,6,24).

**Table 3.** Odds ratios and 95% confidence intervals of determinants of taking children to trained healthcare providers who had fever but no respiratory symptoms in urban Dhaka, Bangladesh (n=1,290)

Variable	Univariate logistic regression OR (95% CI)	Multiple logistic regression OR (95% CI)
Socioeconomic status (by quintile)		
First (poorest)	1.0	1.0
Second	1.8 (1.2-2.7)	1.6 (1.0-2.4)
Third	2.8 (1.9-4.2)	2.2 (1.5-3.2)
Fourth	3.6 (2.3-5.5)	2.6 (1.7-4.0)
Fifth (richest)	7.9 (4.8-13.0)	5.6 (3.4-9.2)
Education of fathers		
No schooling and some primary (reference category)	1.0	1.0
Finished primary and some secondary	1.6 (1.1-2.3)	1.3 (0.9-1.9)
Finished secondary	3.7 (2.7-5.2)	1.8 (1.2-2.5)
Sex of child		
Male	1.3 (1.1-1.6)	1.5 (1.2-1.9)
Presence of decreased level of consciousness		
Yes	4.2 (1.6-10.8)	5.3 (2.0-14.2)
Age (years) of children		
<1	1.6 (1.1-2.3)	1.6 (1.1-2.4)
1-<2	1.7 (1.2-2.3)	1.5 (1.0-2.2)
2-<3	1.2 (1.0-1.8)	1.4 (0.9-2.0)
3-<4	0.9 (0.6-1.3)	1.0 (0.7-1.5)
4-<5 (reference category)	1.0	1.0

CI=Confidence interval;

OR=Odds ratio

Concerns about payment for consultation may be a barrier to seeking qualified healthcare. People from the poorest quintile had incomes below the World Bank-recommended poverty-line (25). Medical consultation with a trained healthcare provider in either of the two hospitals in this study generally costs US$ 0.30-7.00, excluding the purchase of medicines. Purchasing medicines from a drug vendor can save the consultation fee while visiting an untrained healthcare provider is a lower cost one-fee option.

Education of fathers was also found to be associated with seeking healthcare from a trained care provider in studies in settings similar to Bangladesh (9,26,27). In Bangladeshi society, fathers are usually the decision-makers for the family, and it is likely that educated fathers might take their children to qualified providers (28).

Boys were more likely than girls to receive care from qualified healthcare providers. In many parts of South Asia, boys are more likely to be taken, not only to any healthcare providers but also to qualified healthcare providers (20). According to Victoria and colleagues, the inequity in healthcare-seeking by gender contributes to higher mortality rates among girls. Their estimate suggests that if girls and boys had similar mortality rates, child mortality would have dropped by 20% in India, the neighbour of Bangladesh (6). Inequalities in child healthcare by gender in Bangladesh have been reported earlier, and our study extends these findings to care-seeking for febrile illness in urban Dhaka (1,29-31). Although this urban setting has a relatively-low rate of child mortality, disparity in healthcare-seeking by socioeconomic status and gender persists in these communities. Both of these inequalities are important barriers for Bangladesh to achieve the Millennium Development Goal 4 target of reducing childhood mortality.

**Fig.**
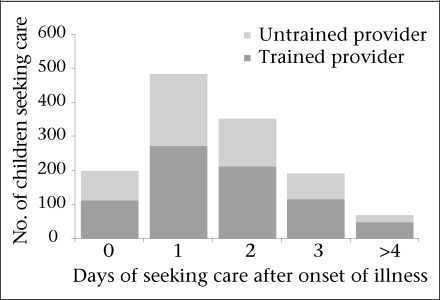
Time of seeking care from different healthcare providers for febrile illness

Additional factors identified as important in seeking healthcare from trained healthcare providers for febrile illness were recognition of the presence of danger-signs and the child's age. If the caregiver was aware of a ‘decreased level of consciousness’, defined as difficulty in staying awake, the children were significantly more likely to be taken to trained healthcare providers. This finding is consistent with findings of several studies that found that the perceived severity of illness influences decision to seek care (20-22,32,33). Even adjusted with wealth and other determinants, people were significantly more likely to take their children to trained healthcare providers if they were aware of danger-signs.

In neonates and young infants, fever can often be the only recognizable sign of a potentially-life-threatening underlying illness. We found that the younger the child, especially infants and children aged less than two years, the more likely that they would be taken to a trained healthcare provider. Results of studies in different low-income countries and data of the BDHS showed similar types of findings (2,21,32,34-36). The highest number of children visiting traditional care providers (homeopaths) was the children aged less than one year, which agrees with findings of studies that homeopathic medicine is a preferable option as it is mild, slow in action, has no side-effects, and, because of its sweet taste, is easy to administer (9,37).

The timing of care-seeking also plays an important role in recovery (38,39). Although we found that 80% of our study caregivers reported seeking care outside the home within two days of onset of fever, 41% of them took their children to untrained providers. A similar pattern of care-seeking could be observed among children who had fever with decreased level of consciousness. Thus, the benefit of promptness might have been diminished because of choice of provider.

In other low-income settings, child health and survival was improved when parents selected qualified healthcare providers rather than non-qualified healthcare providers (40,41). If adequate quality care is available at healthcare facilities, interventions to improve care-seeking behaviour can reduce mortality. Compared to other evaluations in Bangladesh, we found a comparatively-higher rate of care-seeking (59%) from trained healthcare providers in this urban community (7,9). An earlier analysis found that child mortality in this specific community was lower compared to the overall rate of child mortality in Bangladesh (42). One possible explanation is the higher rate of care-seeking from available trained healthcare providers.

Although 59% of the study participants sought care from trained healthcare providers, 41% did not. Despite living in the catchment areas of the two well-functioning paediatric hospitals with outpatient facilities, over one-third of caregivers did not avail themselves of this qualified care, illustrating that physical availability of services alone does not ensure use by all.

### Limitations

An important limitation of the study was that we selected the clusters from those communities where children were routinely admitted to the two hospitals during our study period. Therefore, the study population may not represent communities from where no children were admitted to the study hospitals. Our results may also not be as applicable to other urban communities with less access to large paediatric centres. However, only 13% of the study caregivers sought treatment from the study hospitals. The remaining participants who sought care from trained providers used private practitioners. Metropolitan Dhaka has an abundance of private practitioners compared to public-healthcare facilities; so, it is likely that households in other areas would also use the thriving business of privatized medical care (24).

Another limitation is that, due to the design of the larger study, we did not have information about children who had a severe illness and who might have benefitted from a visit to a healthcare provider but who did not go. In low-income countries, such as Bangladesh, healthcare is not sought from outside the home for a large number of children (7,39). However, we focused on the choice of a qualified versus a non-qualified healthcare provider, which can make a meaningful difference in child survival.

### Conclusions

Although most febrile illnesses are self-limiting, there is a risk that socioeconomic barriers and gender inequalities may result in poor children, especially girls, suffering serious consequences, including death, due to receiving inappropriate care from untrained practitioners (1,2). One short-term approach to reduce this risk is to improve caregivers' recognition of danger-signs relating to febrile illness among under-five children. This knowledge might prompt them to seek healthcare more quickly from a trained healthcare provider. In the short term, a health-education and behaviour-change communication intervention, particularly focused on low-income families, could be designed, implemented, and evaluated. Long-term interventions, such as income generation and literacy programmes, could contribute to fundamental social changes to reduce financial and gender inequalities.

## ACKNOWLEDGEMENTS

The study was funded by the Department of Health and Human Services National Vaccine Program Office (NVPO) through the United States Agency for International Development Global Bureau's Global Research Activity Cooperative Agreement with Johns Hopkins University Bloomberg School of Public Health, and the Government of Bangladesh through IHP-HNPRP. icddr,b acknowledges with gratitude the commitment of the NVPO to the Centre's research efforts. The authors particularly appreciate the suggestions made by Dorothy Southern, Alan Hubbard, and Yushuf Sharker on the manuscript.
